# Editorial for the Special Issue on Advanced Energy Conversion and Storage Microdevices

**DOI:** 10.3390/mi13010138

**Published:** 2022-01-16

**Authors:** Hee-Seok Kim

**Affiliations:** Mechanical Engineering, School of Engineering and Technology, University of Washington Tacoma, Tacoma, WA 98402, USA; heeskim@uw.edu

Advanced energy conversion and storage systems have attracted much attention in recent decades due to the increasing demand for energy and the environmental impacts of non-sustainable energy resources. This has led to the development of renewable energy systems such as photovoltaics, thermoelectrics, piezoelectrics, triboelectrics, batteries, fuel cells, supercapacitors, and many other technologies. Recently, advanced energy conversion and storage systems with a smaller form have been developeding and have been integrated into a wide range of applications: soft electronics, Internet of Things (IoT) devices, personal mobile devices, biomedical systems, and human-interfaced wearable electronics. To drive such compact devices under constrained operating conditions, a sustainable energy supply is essential. An example is the long-term operation of wearable biomedical sensors for continuous monitoring in a daily life. In addition, on-chip micro- and nano-technologies has been integrated into photovoltaic devices and electrocatalytic devices based on low-dimensional structured materials. Advanced energy conversion and storage systems in microdevices are the key to self-powered, compact electronics.

This Special Issue showcases five research articles that explore a wide range of energy-conversion and -storage systems based on piezoelectrics [1], photovoltaics [2], ionic wind pump [3], vibrational magnetic spring [4], and thermoelectrics [5] and two review articles covering the state-of-art technology of soft devices and systems: flexible microsupecapacitors [6] and fiber-based thermoelectrics for wearable electronics [7]. The latter review article has been selected as a feature paper and editor’s choice in Micromachines.

Nonlinearity is beneficial in a piezoelectric vibration energy harvester to produce multiple steady state and deflect the frequency response curves by collecting more energy over a broader frequency range. However, such a configuration seriously affects its energy conversion efficiency. Qichang et al. [1] analyzed how to take full advantage of the nonlinear characteristics to widen the bandwidth of a piezoelectric vibration energy harvester and improve the efficiency and modeled the influence of the inter-permanent magnet torque exerted on the cantilever beam bending of the piezoelectric energy harvesting. The theoretical modeling study showed the effective frequency band of the nonlinear harvester to be 270% wider than that of the linear harvester, which gave rise to obtain more than 0.1 mW in the frequency range of 18 Hz.

The performance of organic solar cells is influenced by the temperature effect, which is caused by film morphology changes and variation in its electrical properties. Chen et al. [2] investigated the influence of the temperature in the range from 77 K to 300 K on the photocurrent and internal/external quantum efficiency of a CuPc/C60 based solar cell, showed that the absorption process has negligible change in this temperature range, and found that exciton diffusion and exciton dissociation play a significant role in affecting the photocurrent of organic solar cells at different temperatures.

Thermal management is a key issue in microelectronics for the failure in heat control which decreases device reliability. One of the common thermal solution is based on forced convection in microelectronics, but the use of a traditional rotary fan to generate airflow is limited due to the space limitations. Ionic wind pump systems have been used due to its small form factor but the inlet blockage effect plays a negative role in the system. Ye et al. [3] presented how the performance of a needle-ring ionic wind pump responds to inlet blockage in different electrical driving modes (direct current), including the flow rate, the corona power, and the energy efficiency. The results show that the performance of small needle-ring ionic wind pumps is sensitive to neither the inlet blockage nor the electrical driving mode, which makes needle-ring ionic wind pumps a viable option for microelectronics.

Vibration-energy-harvesting devices suffer from durability issue due to moving components. For example, a metal spring for a vibration-energy-harvesting device in a high-speed train is often damaged during its operation. Kim [4] applied a non-contact magnetic spring to a vibration-energy-harvesting device using the repulsive force of permanent magnets and conducted experiments in a high-speed train traveling at 300 km/h to study design parameters of the magnetic spring and compare the output power generation of the harvester using the magnetic spring and a conventional metal spring.

Solar radiation is a sustainable source to drive thermoelectric power generators; for example, IoT applications belonging to a sensor grid system. However, most thermoelectric power generators under the solar radiation generate a minor temperature gradient. Oliveira et al. [5] developed a thermoelectric power generator combined with a layer for solar absorption to enhance the temperature gradient across thermoelectric generators. In this study, they developed a compact thermoelectric energy harvester featuring with quasicrystal solar absorber and demonstrated the output performance: 3 V of the output voltage that stored up to 1.38 J in a supercapacitor per day, which led to 29% improvement in energy harvesting compared with that by using conventional black paint coating for the solar absorption.

Flexible on-chip microsupercapacitors (MSCs) have the advantages of a small form factor, light weight, ultrahigh power density, and excellent lifespan, which are beneficial wearable electronics and give it great potential as a stand-alone functional unit in flexible devices. Li et al. [6] summarize the recent progress made in the development of flexible MSCs and their applications in integrated wearable electronics. Current assembly technologies include ink printing, photolithography, screen printing, laser etching, etc., meeting the criteria for scalable fabrication, minimized form factor, and integration into flexible MSCs. This review article includes guidelines on how to improve the electrochemical performance by approaching material design, device construction and electrolyte optimization and discusses the integrated prototypes of flexible MSC-powered systems, such as self-driven photodetection systems and wearable sweat monitoring sensor devices. In addition, future challenges and perspectives of flexible MSC are envisioned.

Flexible energy-harvesting devices with light-weight and self-powering abilities have attracted increasing attention. Flexible generators that capture and convert ambient energy (solar, mechanical, thermal energy, etc.) into electric energy are suitable candidates due to their flexibility and mechanical stability, which enable them to maintain their power generation function during deformation when they fit onto the human skin. Human life is maintained by metabolism, resulting in a continuous heat dispersion from the human body with an energy density of 20 mW/cm${}^{2}$. Thus, the human body is a sustainable heat resource for thermoelectric power generation. Zhang et al. [7] presented a review of state-of-the-art fiber-based thermoelectric material fabrication, device assembly, and its potential applications in temperature sensing, thermoelectric generation, and temperature management. In this mini review, they also shed some light on the potential application in the next generation of wearable electronics and discuss the challenges and opportunities.

The guest editor would like to take this opportunity to thank all the authors for sharing their prestigious work, submitting research and review articles to this Special Issue, and contributing to the field of energy conversion and storage microdevice technologies. I would also like to thank all the reviewers for dedicating their time to providing careful and timely reviews to ensure the quality of this Special Issue.
